# Tortuous Pore Path Through the Glaucomatous Lamina Cribrosa

**DOI:** 10.1038/s41598-018-25645-9

**Published:** 2018-05-08

**Authors:** Bo Wang, Katie A. Lucy, Joel S. Schuman, Ian A. Sigal, Richard A. Bilonick, Chen Lu, Jonathan Liu, Ireneusz Grulkowski, Zachary Nadler, Hiroshi Ishikawa, Larry Kagemann, James G. Fujimoto, Gadi Wollstein

**Affiliations:** 1 Department of Ophthalmology, University of Pittsburgh School of Medicine, UPMC Eye Center, Eye and Ear Institute, Ophthalmology and Visual Science Research Center, Pittsburgh, PA United States; 2 Department of Bioengineering, Swanson School of Engineering, University of Pittsburgh, Pittsburgh, PA United States; 3 NYU Eye Center, NYU Langone Health, Department of Ophthalmology, New York University School of Medicine, New York, NY United States; 4 Department of Electrical and Computer Engineering, Tandon School of Engineering, New York University, New York, NY United States; 5 Department of Neuroscience and Physiology, New York University School of Medicine, New York, NY United States; 6 Department of Biostatistics, University of Pittsburgh School of Public Health, Pittsburgh, PA United States; 7 Department of Electrical Engineering and Computer Science, Massachusetts Institute of Technology, Cambridge, MA United States; 8 Center for Devices and Radiological Health, Food and Drug Administration, Silver Spring, MD United States

## Abstract

The lamina cribrosa is a primary site of damage in glaucoma. While mechanical distortion is hypothesized to cause reduction of axoplasmic flow, little is known about how the pores, which contains the retinal ganglion cell axons, traverse the lamina cribrosa. We investigated lamina cribrosa pore paths *in vivo* to quantify differences in tortuosity of pore paths between healthy and glaucomatous eyes. We imaged 16 healthy, 23 glaucoma suspect and 48 glaucomatous eyes from 70 subjects using a swept source optical coherence tomography system. The lamina cribrosa pores were automatically segmented using a previously described segmentation algorithm. Individual pore paths were automatically tracked through the depth of the lamina cribrosa using custom software. Pore path convergence to the optic nerve center and tortuosity was quantified for each eye. We found that lamina cribrosa pore pathways traverse the lamina cribrosa closer to the optic nerve center along the depth of the lamina cribrosa regardless of disease severity or diagnostic category. In addition, pores of glaucoma eyes take a more tortuous path through the lamina cribrosa compared to those of healthy eyes, suggesting a potential mechanism for reduction of axoplasmic flow in glaucoma.

## Introduction

The lamina cribrosa (LC) has been identified as one of the primary sites of damage in glaucoma, the second leading cause of blindness worldwide^1^. The LC, a connective tissue meshwork located deep in the optic nerve head, contains pores through which all retinal ganglion cell axons pass on their way to the brain. One of the defining features of glaucoma is a reduction of axoplasmic transport^2–4^. Experimental models have demonstrated an accumulation of axonal material, such as mitochondria, at the level of the LC^4,5^.

It is hypothesized that distortion of the LC may impede axoplasmic transport^3,6,7^. These distortions are characterized by increased tortuosity of the axons, which prevent the proper flow. However, despite this hypothetical mechanism, there have been few studies assessing pore pathways through the LC *in vivo*^8^.

While characterizing the LC with histology would allow the tracing of individual axonal bundles with depth, there are certain advantages to characterizing the LC pore paths *in vivo*. *In vivo* characterization eliminates artifacts that could potentially be caused by cryosectioning of the LC, which may alter the microstructure and the path of the pores. In addition, *in vivo* studies do not require registration of adjacent histology section to create a 3D reconstruction. Characterization of individual LC pore paths is now possible due to advances in optical coherence tomography (OCT) technology in the analysis of LC microstructure. OCT has the advantage of acquiring detailed 3D imaging of the LC without external perturbation that could alter its microstructure. However, it is important to recognize that unlike histology, *in vivo* characterization is limited in the region of the LC visible by OCT. While the limited transverse resolution of conventional OCT (~15–20 μm) does not permit characterization of individual axons, it does allow the characterization of the path of pores which reflects the path of the axons. These pore paths can serve as a surrogate for the path of axons, as previous histology studies have demonstrated that the vast majority of axons follow their LC pore path^8^.

Many recent studies using OCT investigate *in vivo* how the LC is different in glaucoma subjects compared to healthy controls^9–11^. Despite the ability to image the LC, many *in vivo* studies have been limited to the assessment of the LC macrostructure, such as total thickness and anterior surface features^9,12^. A number of groups, including ourselves, have published methods of quantifying the LC microstructure, the complex beams and pore that make up the LC, in 3D and *in vivo*^13–15^. These automated segmentation algorithms are used to quantify LC microstructure and identify differences between healthy and glaucoma subjects^13,16^. The purpose of this study is to investigate *in vivo* whether the pores of glaucomatous eyes take a more tortuous path through the LC compared to healthy eyes. We utilize our single pore tracing analysis to trace and quantify in 3D the individual pores in order to characterize their path through the LC.

## Methods

### Subjects

This study was conducted in accordance with the tenets of the Declaration of Helsinki and the Health Insurance Portability and Accountability Act. The institutional review board of the University of Pittsburgh approved the study. All subjects gave written informed consent prior to participating in the study.

One hundred and five eyes were sequentially recruited, from the University of Pittsburgh Medical Center (UPMC) Eye Center, out of which 87 eyes (16 healthy, 23 glaucoma suspect, 48 glaucoma eyes; 70 subjects) had sufficient image quality to allow further analysis. The subjects underwent a comprehensive ophthalmic examination, including slit lamp biomicroscopy, intraocular pressure measurement, visual field testing (Humphrey visual field analyzer, Zeiss, Dublin, CA; Swedish interactive threshold algorithm 24–2) and OCT scanning. Healthy eyes were characterized as having normal appearance of the optic nerve head (ONH) and retinal nerve fiber layer (RNFL), and full visual fields without any previous history of retinal diseases or glaucoma. Glaucomatous eyes were classified by clinical examination findings characteristics for glaucoma (ONH abnormality: global rim thinning, rim notch, or disc hemorrhage; RNFL defect) accompanied with reproducible typical visual field loss. Glaucoma suspects had suspicious ONH appearance (as described above), RNFL defect, or the contralateral eye of a unilateral primary open angle glaucoma subject.

### Imaging

The LC was scanned using a swept source OCT imaging device with 100 kHz scan rate and a 1050 nm central wavelength^17^. A 3.5 mm × 3.5 mm × 3.64 mm volume centered on the optic nerve head was taken, with the focus set to the level of the LC. A total of 400 × 400 A-scans were performed for each volume, with a spacing of 8.75 μm between A-scans. To eliminate motion artifacts, two orthogonally oriented scan volumes (one 3D volume oriented horizontally and one 3D volume oriented vertically) were registered using a previously described algorithm^18^.

### Tracking analysis

LC pore microstructure was automatically segmented using a previously described method^13,19^. The posterior LC was defined by a drop-off in reflectivity of the LC on OCT. The images were made isotropic (4.065 μm/pixel in all dimensions) prior to proceeding with the analysis.

**Figure 1:**
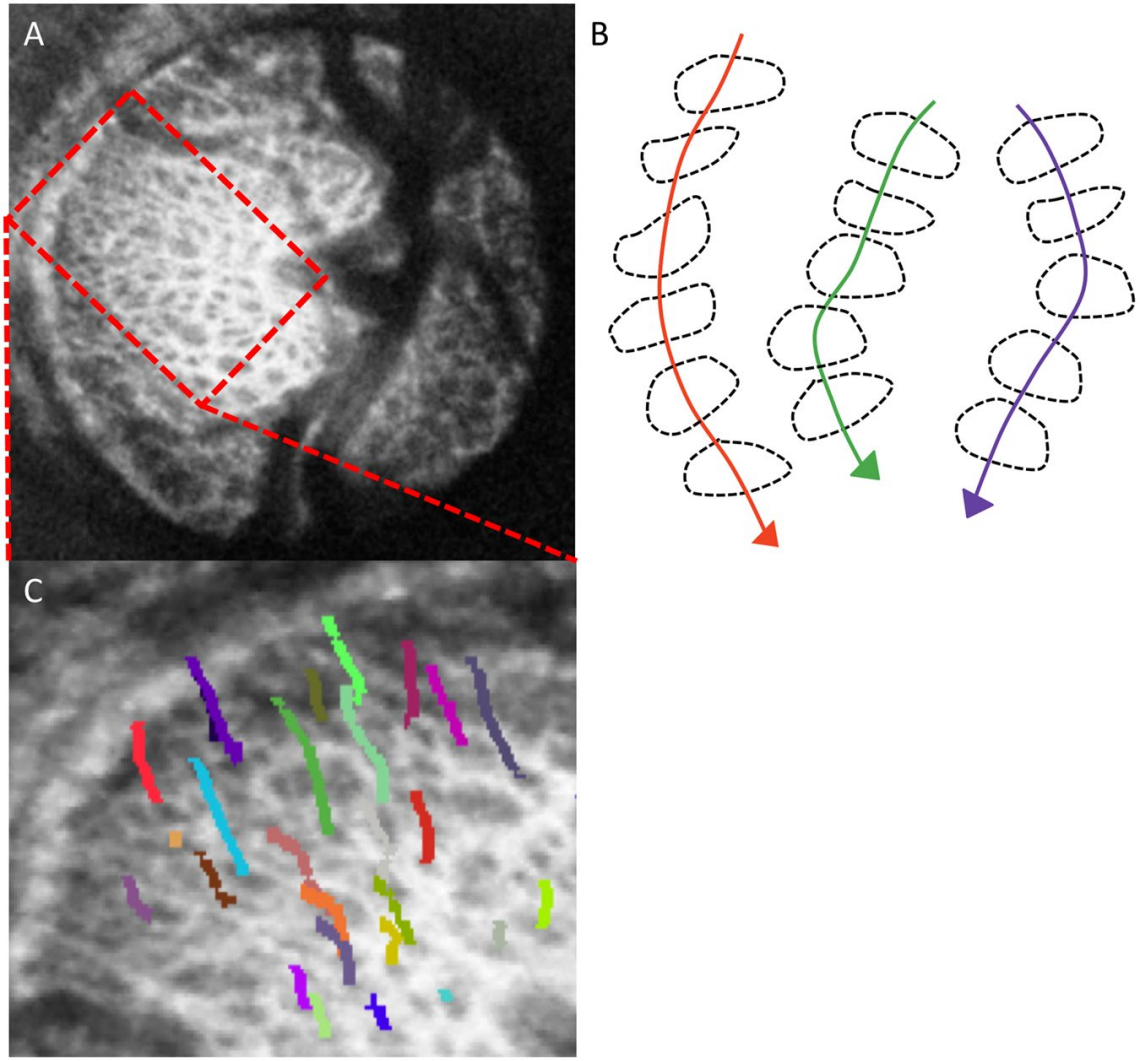
Pore path tracing. (**A**) Enface view of the lamina cribrosa. (**B**) Pore paths were traced with respect to depth via the centroid of the segmented pores. (**C**) 3D view of pore path tracing from a subset of pores in a single eye (27 out of 81).

Pore path was traced automatically in ImageJ using a particle tracking algorithm (MTrack2; implemented by Nico Stuurman), which allowed tracing of individual pores through the LC volume. The tracing was done from one C-mode to the next, using the centroid of the pores. To improve identification of pore paths from one C-mode to the next, the tracings were constrained such that pores could move a maximum of 20 μm in the transverse direction when advancing 4 μm into the next C-mode stack. This value was chosen since it was smaller than a typical pore diameter, ensuring that adjacent pores were not selected for tracing. Only pores that could be traced for a distance of at least 60 μm in the Z-direction were selected for analysis. The values were chosen as it represented a balance from capturing all the pore paths versus having inaccurately labeled pore paths. All pores paths were viewed in 3D to ensure proper tracing (Fig. 1) prior to inclusion in the analysis. The average depth of pores tracked in each eye was recorded. Bifurcations were treated as new pore paths to be analyzed.

### Tracking Analysis Validation

10 randomly chosen pore paths from 20 different eyes (total of 200 pore paths) were chosen to compare against manual tracing of pore paths. A human observer (BW) traced the path from anterior LC to posterior LC and the results of the tracing were compared to the automated tracing.

### Assessment of pore trajectory relative to the disc

**Figure 2:**
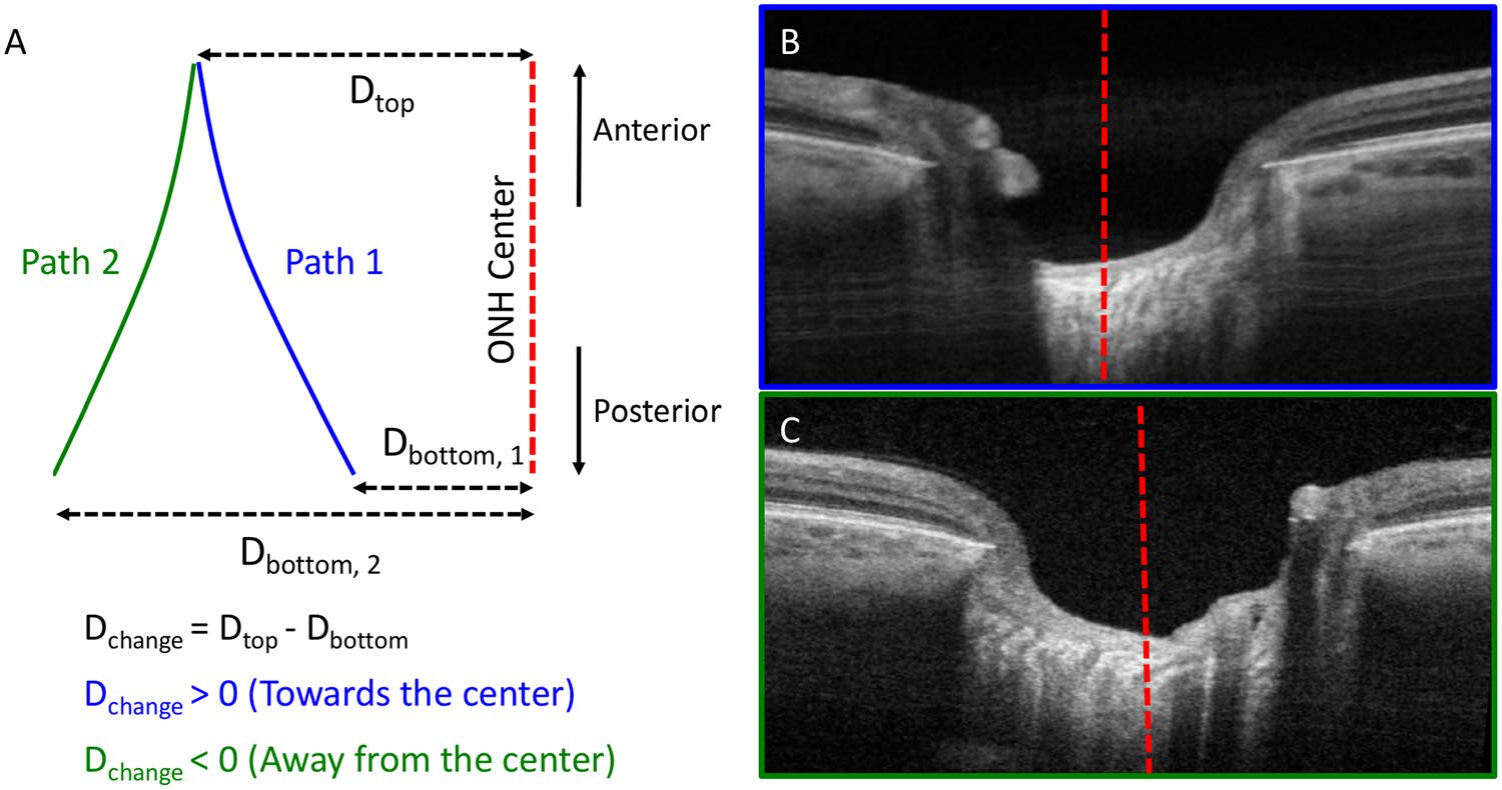
Identification of pore path relative to the disc. (**A**) Method of identification of pore path relative to the central disc (dotted red line). Positive distance change (path 1; blue) indicated convergence towards the center, while negative distance change (path 2; green) indicated convergence away from the center. (**B**) Example of LC identified to have pores going towards the disc center (red dotted line) and (**C**) away from disc center.

To fully characterize pore trajectory in the LC, we analyzed the pores path relative to the center of the optic nerve. The center of the optic nerve was defined as the centroid of the Bruch membrane opening (BMO). The distance the pore path moved towards the center of the optic nerve was computed using the method described in Fig. 2. We defined a positive distance value as situations where the LC pore path was closer to the center of the optic nerve as it traversed the LC anteriorly to posteriorly, indicating convergence of pores towards the center. A negative distance value implied that the pore path was more peripherally located as it traversed the LC anteriorly to posteriorly. The change in distance was computed for each pore and averaged across all pores for a given eye. This distance was normalized based on the depth of pore path, as it was expected that pores that were traced for longer distance may have more movement in the X-Y direction.

### Assessment of pore tortuosity

Pore tortuosity was assessed by dividing the distance traveled by the pore centroid by a straight line connecting the anterior pore to the posterior pore. All tortuosity values were restricted to 1, or greater. A value near 1 indicated that the pore went in a straight line between the anterior and posterior pore, while a value greater than 1 indicated that the pore took a tortuous path.

### Statistical analysis

One sample Kolmogorov-Smirnov test was used to determine each eye’s tortuosity distribution in order to verify if a global average summary for each eye was appropriate due to the normality of the distribution. Linear mixed effects analyses were used to determine how (1) average pore depth tracked, (2) pore movement towards the center and (3) how pore tortuosity was affected by diagnosis and disease severity as assessed by visual field mean deviation (VF MD). The linear mixed effects models were random intercept models, with the random effect of each eye accounting for the expected autocorrelation between eyes.

## Results

### Study Demographics

Study population characteristics are summarized in Table 1. The average age of healthy subjects (45.6 ± 12.5 years) was significantly younger than that of glaucoma suspects (60.7 ± 8.0) and glaucoma subjects (68.3 ± 14.4). There was no statistically significant difference in VF MD between healthy subjects (−0.85 ± 1.26 dB) and glaucoma suspects (−0.09 ± 0.85). Glaucoma subjects (−7.62 ± 6.94) had a significantly lower VF MD compared to healthy subjects. Finally, there was no statistically significant difference in percentage of visible LC between diagnostic categories.

**Table 1:** Study population characteristics.

	Healthy	Glaucoma suspect	Glaucoma	GS vs H	GL vs H
Subjects (eyes)	12 (16)	18 (23)	40 (48)		
Age (years)	45.6 ± 12.5	60.7 ± 8.0	68.3 ± 14.4	<0.01	<0.01
VF MD (dB)	−0.85 ± 1.26 dB	−0.09 ± 0.85	−7.62 ± 6.94	0.72	<0.01
Visible LC (%)	23.5 ± 6.2	24.0 ± 8.0	28.1 ± 7.3	0.88	0.05

Number of eyes, age, visual field mean deviation (VF MD) and percentage of visible lamina cribrosa (LC) seen on OCT were listed for the different diagnostic categories (healthy – H, glaucoma suspect – GS, glaucoma – GL).

### Tracking Analysis Validation

Using manual tracing as gold standard, the percentage of pores accurately traced by the computer was 98.5% (3 errors out of 200 pores traced). All three errors stemmed from the segmentation analysis causing occasional merging of adjacent pores, which caused the pore path to be located between the pores, instead of on the pores.

### Depth of LC pore path tracking

The LC pore paths were tracked for average distance of 157 ± 16 μm, 159 ± 15 and 140 ± 14 for healthy, glaucoma suspect and glaucoma subjects, respectively. There was a statistically significant smaller depth tracked between glaucoma and healthy (p = 0.002) and glaucoma and glaucoma suspects eyes (p < 0.001).

### Normality of pore distance change and pore tortuosity

Only 2 out of 87 eyes had non-normal distributions of pore tortuosity, and only 1 out of 87 had a non-normal distribution of pore distance change towards the center. Therefore, we used an average of the distributions within each eye as a summary value.

### Assessment of pore trajectory relative to the optic nerve

**Figure 3:**
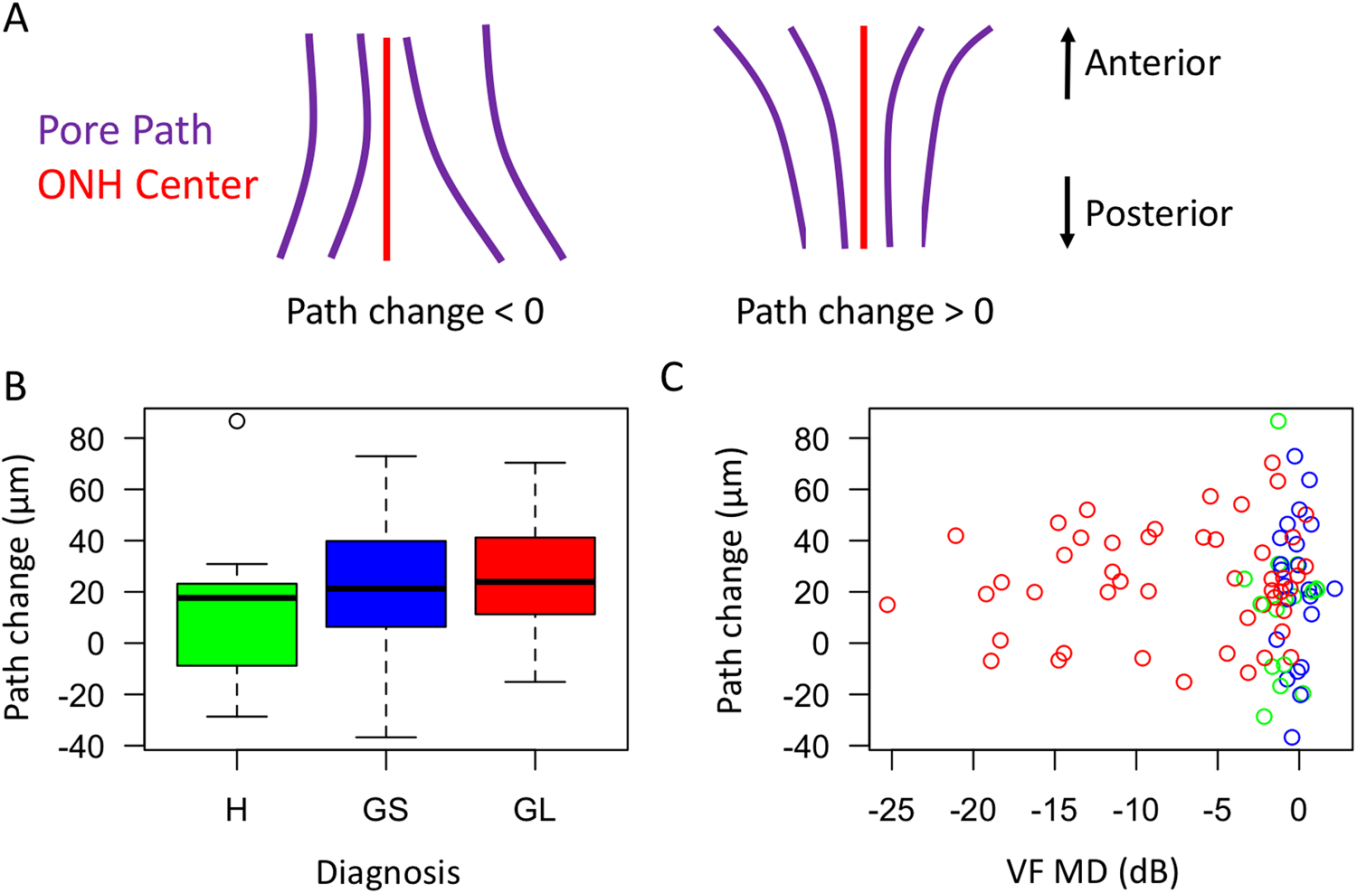
Assessment of pore path relative to disc. (**A**) Schematic demonstration of examples of negative path change (pores move away from disc center going from anterior to posterior) and positive path change (pores move towards the disc center). (**B**) Boxplot of pore path change with respect to diagnosis (H – healthy, GS – glaucoma suspect, GL – glaucomatous eyes) and (**C**) visual field mean deviation.

Average pore paths within the LC traversed toward the optic nerve center posteriorly for a distance of 21.4 ± 23.0 μm between the anterior and posterior surfaces of the LC (p < 0.001). For each diagnostic category (Fig. 3), the average convergence were 13.6 ± 27.2 μm (healthy), 21.4 ± 27.3 (glaucoma suspect), and 24.0 ± 21.1 (glaucoma). There was no statistically significant difference in average pore path change amongst the diagnostic categories before or after normalizing by the average depth of pores traced. Therefore, all data regarding pore trajectory shows non-normalized pore path changes.

### Assessment of pore tortuosity

**Figure 4:**
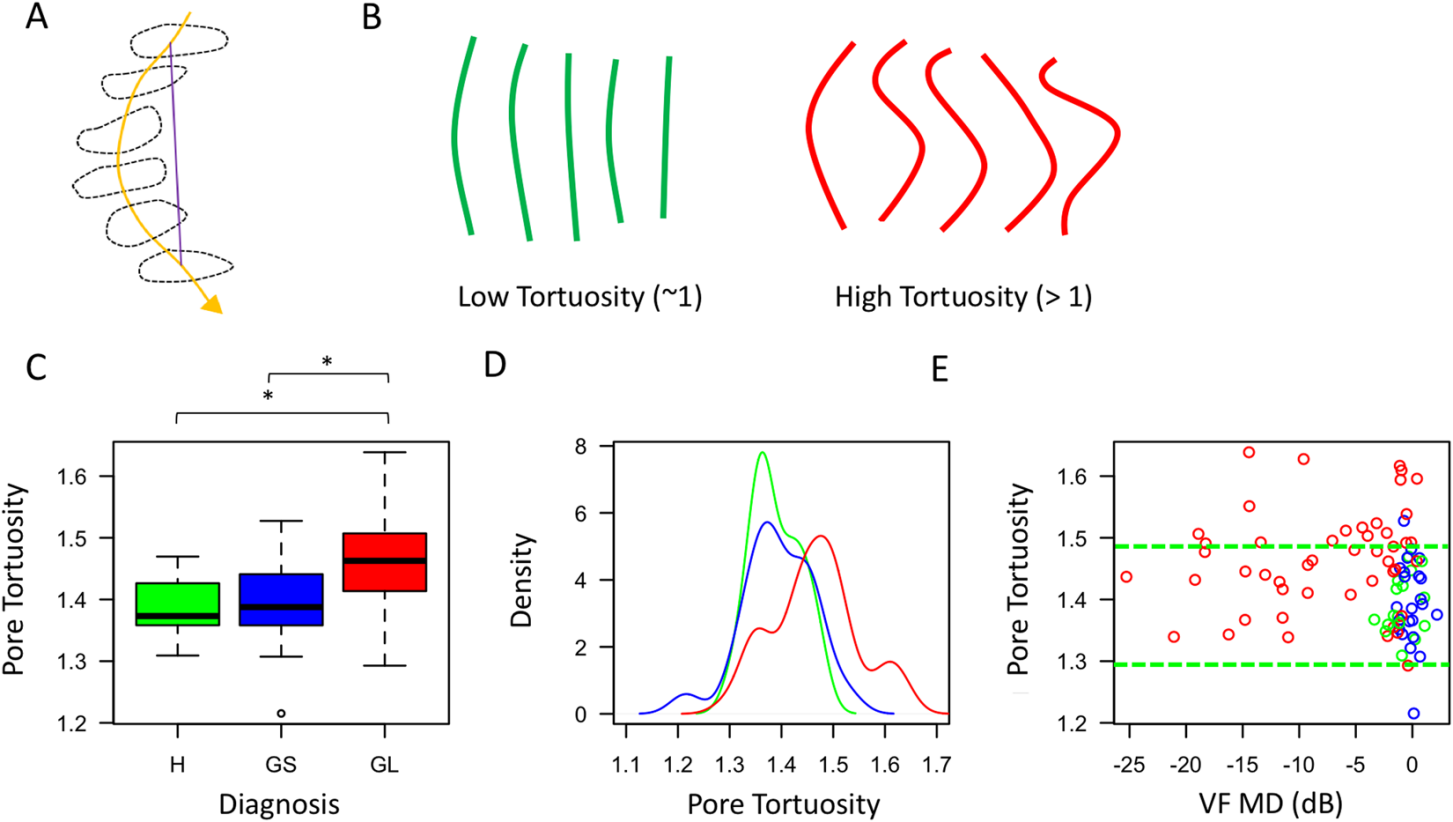
Assessment of pore tortuosity. (**A**) Pore tortuosity was defined by dividing the distance traveled by the pore centroid (yellow line) by the shortest distance between the top and bottom pore (purple line). (**B**) Schematic showing examples of low tortuosity (green) and high tortuosity (red). (**C**) Boxplot of tortuosity as a function of diagnosis (H – healthy, GS – glaucoma suspect, GL – glaucomatous eyes). (**D**) Probability density distribution of pore tortuosity as a function of disease (Green – healthy, Blue – glaucoma suspect, Red – glaucomatous eyes). (**E**) Pore tortuosity as a function of visual field mean deviation, with the green lines denoting the 95% confidence interval of the healthy subjects.

Pore tortuosity in glaucomatous eyes (1.46 ± 0.08) was significantly higher than in healthy (1.39 ± 0.05, p < 0.01) and glaucoma suspect eyes (1.39 ± 0.07, <0.01) (Fig. 4C). Glaucomatous eyes also had larger variance compared to healthy and glaucoma suspect (p < 0.01, <0.01, respectively) (Fig. 4D). Glaucoma severity, as determined by baseline VF MD, did not affect the pore tortuosity (Fig. 4E).

## Discussion

In this study, we investigated the LC pore path *in vivo* as a surrogate of the ganglion cell axonal pathway. We identified higher tortuosity and larger variability in the pore paths of glaucomatous eyes compared with healthy and glaucoma suspect eyes. These findings are the first evidence *in vivo* that the spatial pathway of pores traversing the LC corroborate with histological evidence of distorted axons in glaucomatous eyes. While our cross-sectional study design cannot argue for causation, it does suggest a potentially critical component of axonal insult either as direct contribution to glaucomatous damage, or as a consequence of the glaucomatous process, such as remodeling.

### Depth of LC pore path tracking

As expected, there are significant differences between diagnostic category with respect to the average depth of LC pores tracked. Previous histological^20^ and *in vivo* studies^21–23^ have demonstrated significantly thinner LC in glaucoma subjects, and have found that thinner LC measurements were associated with worst VF MD. Therefore, the decreased depth tracked in glaucoma subjects can be explained by the fact that glaucoma subjects have a thinner LC. However, the depth LC pores were tracked was approximately 20% lower than the average LC thickness reported by others *in vivo* studies^21,23^. This is likely due to the fact that while it is possible to see the posterior LC on B-scans, it is difficult to achieve adequate imaging of the posterior LC to perform segmentation on LC microstructure. Therefore, it can be expected that the average depth tracked will be lower than that of the LC thickness reported by analyses of OCT macrostructure on individual B-scans.

### Pore trajectory relative to the optic nerve

Regardless of diagnostic categories, LC pores tended to converge towards the center of the optic nerve while moving from the anterior to posterior LC. This finding can be explained by retinal ganglion cell axons in most eyes being bottlenecked at the level of the canal opening, causing the axons to converge towards the center. Alternativly, this can be the result of retrolaminar myelin coverage of the axons. Furthermore, there was no statistically significant difference in the convergence between the diagnostic categories, even after accounting for the depth of LC pore path traced. The current resolution and signal penetration of OCT does not permit identification of the myelin coverage or retrolaminar axonal path and therefore further investigation is warranted to clealrly allucidate the cause.

### Pore tortuosity

While a number of histological studies have demonstrated the blockage of axoplasmic transport at the level of the LC^4,5,24^, this study is the first to identify *in vivo* a potential mechanical reason for the blockage of flow. The increased tortuosity in glaucoma subjects may reduce axoplasmic flow and contribute to the symptoms of glaucoma. The increased tortuosity is perhaps a result of non-uniform strain and stress experienced by the LC^25,26^. It is likely that these factors cause the LC pores to experience strains in different directions as the axons traverse the LC.

A surprising finding of this study is that elevated tortuosity was noted even in eyes with very early disease (Fig. 4E). Our initial expectations were to find a progressive increase in tortuosity as glaucomatous damage worsened. However, it is possible that increased tortuosity is one of the first steps in glaucoma pathogenesis, which leads to axonal loss as axoplasmic flow is reduced. Additional longitudinal studies are required to elucidate whether the increased tortuosity is an early marker for disease, or even predisposes patients to disease. In addition, there appears to be two categories of glaucoma subjects based on pore tortuosity: those who remain at the tortuosity level of healthy subjects (within the dashed green lines, Fig. 4E) and those outside of it. It can be speculated that glaucomatous eyes that overlap with the tortuosity of healthy eyes already exhibit the outcome of remolding that aims to restore LC integrity in the presence of elevated stress and shears. Further studies are required to elucidate how these groups differ and determine the effect of remodeling.

There are several limitations to the study. First, there is only partial visibility of the LC with OCT due to signal obstruction by the major vasculature within and above the optic nerve. This is a well-known and inevitable limitation for all OCT-based investigations of deep optic nerve head structures. However, as we pool together information from the entire study cohort we expect better representation of most LC, thus reducing the possibility of inadvertent bias. There is also no statistically significant difference in percent of visible LC between the different diagnostic categories, reducing our concerns for potential bias. In general, the central part of the LC will be visible in all subjects. Another limitation is that the age of our healthy subjects is younger than that of the glaucoma subjects. Further investigation with a larger cohort of healthy subjects is required to identify the role of age on pore path and tortuosity. Finally, we cannot discern if the increased tortuosity we detected in glaucomatous eyes was present before the manifestation of glaucomatous signs and symptoms, imposing a mechanical stressor on otherwise healthy neural tissue, instigating glaucomatous changes as part of the pathogenesis of disease, or if increased tortuosity occurred as part of a remodeling process associated with other changes in an ongoing disease process. A longitudinal study in a group of otherwise healthy eyes, but at risk for glaucoma, is needed.

In conclusion, we demonstrated *in vivo* that the pore path within the LC of glaucomatous eyes is more tortuous than in healthy and glaucoma suspect eyes. This finding can explain an important mechanism for axonal damage that is ultimately associated with the glaucomatous process.

## Electronic supplementary material

Supplemental figure legend

Supplemental Video 1 
