# Supplementary material for: Tortuous Pore Path Through the Glaucomatous Lamina Cribrosa
Source: Sci Rep. 2018 May 8;8:7281. doi: 10.1038/s41598-018-25645-9 (PMC5940889; doi:10.1038/s41598-018-25645-9)
Supplement: Supplementary file 1 — Supplemental figure legend [file 41598_2018_25645_MOESM1_ESM.docx]

**Tortuous Pore Path Through the Glaucomatous Lamina Cribrosa**

**Authors**: Bo Wang^1,2^, Katie A. Lucy^3^, Joel S. Schuman^3,4,5^, Ian A. Sigal^1,2^, Richard A. Bilonick^1,6^, Chen Lu^7^, Jonathan Liu^7^, Ireneusz Grulkowski^7^, Zachary Nadler^1^, Hiroshi Ishikawa^3^, Larry Kagemann^3,8^, James G. Fujimoto^7^, Gadi Wollstein^3^

**Affiliation:** ^1^Department of Ophthalmology, University of Pittsburgh School of Medicine, UPMC Eye Center, Eye and Ear Institute, Ophthalmology and Visual Science Research Center, Pittsburgh, PA, United States.

^2^Department of Bioengineering, Swanson School of Engineering, University of Pittsburgh, Pittsburgh, PA, United States.

^3^NYU Eye Center, NYU Langone Health, Department of Ophthalmology, New York University School of Medicine, New York, NY, United States.

^4^Department of Electrical and Computer Engineering, Tandon School of Engineering, New York University, New York, NY, United States.

^5^Department of Neuroscience and Physiology, New York University School of Medicine, New York, NY, United States.

^6^Department of Biostatistics, University of Pittsburgh School of Public Health, Pittsburgh, PA, United States.

^7^Department of Electrical Engineering and Computer Science, Massachusetts Institute of Technology, Cambridge, MA, United States.

^8^Center for Devices and Radiological Health, Food and Drug Administration, Silver Spring, MD, United States.

**Supplemental Video 1**: Video of 3D view of pores traced from a subset of pores in a single eye. Each different color represents a different pore path.
